# Mosaic Intronic *NIPBL* Variant in a Family With Cornelia de Lange Syndrome

**DOI:** 10.3389/fgene.2018.00255

**Published:** 2018-07-13

**Authors:** Natalia Krawczynska, Alina Kuzniacka, Jolanta Wierzba, Ilaria Parenti, Frank J. Kaiser, Bartosz Wasag

**Affiliations:** ^1^Department of Biology and Medical Genetics, Medical University of Gdańsk, Gdańsk, Poland; ^2^Laboratory of Clinical Genetics, University Clinical Centre, Gdańsk, Poland; ^3^Department of Pediatrics, Hematology and Oncology, Medical University of Gdańsk, Gdańsk, Poland; ^4^Department of General Nursery, Medical University of Gdańsk, Gdańsk, Poland; ^5^Section for Functional Genetics, Institute of Human Genetics, Lübeck, Germany

**Keywords:** Cornelia de Lange syndrome, CdLS, *NIPBL* gene, mosaic variant, family case, deep sequencing

## Abstract

Cornelia de Lange Syndrome (CdLS) is a well described multiple malformation syndrome caused by alterations in genes encoding subunits or regulators of the cohesin complex. In approximately 70% of CdLS patients, pathogenic *NIPBL* variants are detected and 15% of them are predicted to affect splicing. Moreover, a large portion of genetic variants in *NIPBL* was shown to be somatic mosaicism. Here we report two family members with different expression of the CdLS phenotype. In both individuals, a c.869-2A>G (r.869_1495del) substitution was detected, affecting a conserved splice-acceptor site. Deep sequencing revealed the presence of somatic mosaicism in the mother. The substitution was detected in 23% of the sequencing reads using DNA derived from blood samples and 51% in DNA from buccal swabs. The analysis of blood DNA of the son excluded the presence of somatic mosaicism. Correlation of molecular and clinical data revealed that various distribution of genetic alteration in different cell types had an impact on the expression of observed clinical features in both individuals.

## Introduction

Cornelia de Lange syndrome (CdLS; OMIM: 1227470, 300590, 610759, 614701, and 300882) is a rare genetic disorder characterized by a highly variable clinical presentation. It is caused by genetic alterations in *NIPBL*, *SMC1A*, *SMC3*, *HDAC8*, and *RAD21* (Krantz et al., 2004; Tonkin et al., 2004; Musio et al., 2006; Deardorff et al., 2007, 2012a,b). To date, genetic alterations in *NIPBL* have been detected in approximately 70% of CdLS patients (OMIM: 608667) (Nizon et al., 2016). Genetic variants affecting gene splicing account for 15% of *NIPBL* mutations (Mannini et al., 2013; Teresa-Rodrigo et al., 2016).

Castronovo et al. (2010) reported the first patient with a mosaic alteration in *NIPBL*. Additional studies have shown that somatic mosaicism is very frequent in CdLS patients (Huisman et al., 2013). More recently, familial mosaic cases have been reported (Parenti et al., 2016).

Here, we report a correlation of clinical and molecular data of two family members with a genetic variant in *NIPBL* affecting a conserved splice-acceptor site. Both Individuals were previously reported as patients harboring a heterozygous germline mutation (Teresa-Rodrigo et al., 2014). However, additional molecular analysis revealed this variant to be somatic mosaicism in the mother, thus explaining her milder CdLS phenotype in comparison to her son, who is not mosaic for the disease-causing variant.

## Background

### Case Report

**FIGURE 1:**
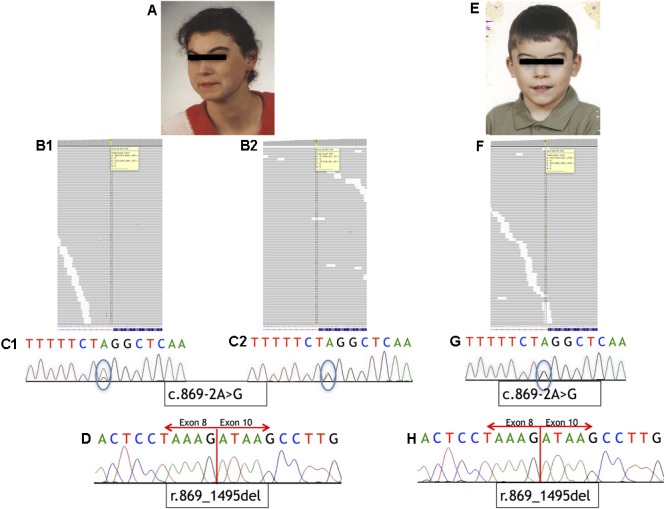
Phenotype of the patients and sequencing results. **(A–D)** Analysis of Individual 1. **(A)** Phenotype of Individual 1. **(B)** IGV view of NGS sequence analysis of exon 9 – intron 8 boundaries of the *NIPBL* gene. **(B1)** A splice-site variant identified in DNA from blood sample in *NIPBL* (alternative allele shown in orange) in 23% of the reads (gray bars). **(B2)** A splice-site variant identified in DNA from buccal swab sample in *NIPBL* (alternative allele shown in orange) in approximately 51% of the reads (gray bars). **(C1)** Sequence analysis of DNA from blood sample of *NIPBL* gene. The mutation is marked with a circle. **(C2)** Sequence analysis of DNA from buccal swab sample of *NIPBL* gene. The mutation is marked with a circle. **(D)** Sequence analysis of cDNA *NIPBL* gene. Panel exhibit an in-frame deletion of exon 9 of *NIPBL* gene in Individual 1. **(E–H)** Analysis of Individual 2. **(E)** Phenotype of Individual 2. **(F)** IGV view of NGS sequence analysis of exon 9 – intron 8 boundaries of the *NIPBL* gene. A splice-site variant identified in DNA from blood sample in *NIPBL* (alternative allele shown in orange) in 46% of the reads (gray bars). **(G)** Sequence analysis of DNA *NIPBL* gene. The mutation is marked with a circle. **(H)** Sequence analysis of cDNA *NIPBL* gene. Panel exhibit an in-frame deletion of exon 9 of *NIPBL* gene in Individual 1.

Individual 1 is a 41 years old female admitted to hospital at the age of 36 years. She was the second born child of healthy, unrelated, mid-30 parents with no family history of congenital defects. She was born at 38 weeks of uncomplicated gestation. Her birth weight, birth length, and the head circumference were 2900 g (-0.65 SD), 48 cm (-1.04 SD), and 30 cm (-3.31 SD), respectively. She presented with hirsutism and mild musculoskeletal anomalies were also noted including small hands and feet, together with bilateral clinodactyly of the fifth finger. A mild craniofacial dysmorphism was also observed (**Figure 1A** and **Table 1**).

**Table 1:** Features of two related CdLS patients.

		Individual 1 CdLS02M	Individual 2 CdLS02
	Age	41 years	8 years
	Gender	Female	Male
System	Specific feature		
Facial dysmorphism	Synophrys	+	+
	Long eyelashes	+	+
	Short nose	+	+
	Anteverted nostrils	+	+
	Long philtrum	+	+
	Broad or depressed nasal bridge	+	+
	Thin lips with downturned corners	+	+
	Low set ears	+	+
Growth	Birth weight (pregnancy week)	2900 g (38 hbd)	2870 g (38 hbd)
	Birth length	48 cm	49 cm
	Birth head circumference	30 cm	32 cm
	Postnatal microsomy	+	+
Development	Developmental delays or intellectual disability	+	+
	Learning disability	+	+
Behavior	Attention deficit disorder	-	+
	Hyperactivity	-	+
	Self-injurious behavior	-	+
	Autistic-like features	-	+
Musculoskeletal	Small hands and feet	+	+
	Fifth finger clinodactyly	+	+
	Short first knuckle/proximally placed thumb	+	+
Cardiac Gastrointestinal	Type of malformation		
	Poor feeding	+	+
	GER	-	+
	Constipation	-	+
Skin	Hirsutism	+	+
	Deafness or hearing loss	+	+ (20 dB)
Urogenital	Cryptorchidism	-	+


*“+,” feature present; “-,” feature not present; GER, gastro-oesophageal reflux; hbd, week of pregnancy.*

At infancy she exhibited a slight hypertonia, poor sucking reflex and a poor weight gain. At the age of 2 years she started to suffer from constipation. She was able to sit unsupported around the age of 8 months, walk independently around the age of 13 months. She started to pronounce syllables around the age of 2 years, used simple words around the age of 3 years, but only at the age of 4 years she started to speak with full sentences. She finished regular primary and secondary school with a great help from her parents. She is a very nice person, easily making contact with other people. Currently, with her parents’ help, she is taking care of her son (Individual 2). Her first pregnancy terminated with a miscarriage at the 23rd week of gestation, but prenatal genetic tests were not performed in that case. All clinical features are summarized in **Table 1**.

Individual 2, the 8 years old son of Individual 1, was the first-born child of 33-year-old mother and 34-year-old healthy father. The pregnancy was complicated by gestational diabetes, regulated by diet and insulin. He was born at 38 weeks of gestation by cesarean section due to placental insufficiency. His birth weight, birth length, and the head circumference were 2870 g (-1.01 SD), 49 cm (-0.91 SD), and 32 cm (-2.07 SD), respectively. His hands and feet were small but not malformed. Bilateral clinodactyly of the fifth finger was evident. He presented with hirsutism and mild craniofacial dysmorphism (**Figure 1E** and **Table 1**).

He demonstrated cryptorchidism and inguinal hernia. In addition, transient hypoglycemia, icterus, and lack of the sucking reflex were observed. During infancy, he had clinical features compatible with gastroesophageal reflux disease (GERD), although it could not be confirmed by pH-metry and endoscopy. The audiometry and auditory brainstem response (ABR) performed at the age of 1 year revealed a mild bilateral sensorineural hearing loss (20 dB). Hand roentgenograms performed in the age of 3 years revealed a delayed bone age.

The patient exhibited motor and language delays. He was able to sit unsupported around the age of 12 months and to walk independently around the age of 18 months. He started to speak syllables around the age of 2 years, simple words around the age of 4 years. At the present time, he still has limited speech abilities. He presents with autistic-like features, some episodes of aggression, and self-injurious behavior. All clinical features are summarized in **Table 1**.

### Materials and Methods

Both blood samples and buccal swabs were obtained after written informed consent from both individuals. Genomic DNA was isolated from peripheral blood lymphocytes and oral mucosa epithelial cells using Genomic DNA from blood kit and Genomic DNA from tissue (Macherey-Nagel) according to the manufacturer’s protocols.

Targeted gene-panel via deep sequencing analysis was performed by using NimbleGen SeqCap EZ HyperCap (Roche Diagnostics) and MiSeq (Illumina). The custom designed panel was spanning 92 kb of the selected genomic sequences including: *NIPBL*, *SMC1A*, *SMC3*, *RAD21*, *HDAC8*, *STAG1*, *SGOL1*, *PDS5A*, *PTTG1*, *TAF6*, *ESCO2*, *WAPAL*, *CDCA5*, *KMT2A*, *DDX11*, *ESPL1*, *PDS5B*, *PLK1*, *AURKB*, *ESCO1*, *MAU2*, *ATRX*, *STAG2*, and *RECQL4* genes. Probes were designed to enrich exons and 25 bp of flanking introns. The analysis was performed using IGV (Broad Institute) and Alamut (Interactive Biosoftware) softwares. The nomenclature of the alteration was based on *NIPBL* mRNA sequence NM_133433.3 according to the HGVS recommendations (den Dunnen et al., 2016). Presence of *NIPBL* alteration was confirmed by independent PCR followed by bidirectional Sanger sequencing.

Total RNA was extracted from blood samples of Individuals 1 and 2 using the Total RNA Mini (A&A Biotechnology). GoScript Reverse Transcriptase (Promega) was applied for cDNA synthesis. For both Individuals, exons 8–10 of *NIPBL* gene were amplified. PCR products were excised from agarose gel and bidirectionally sequenced. PCR products were sequenced using BigDye Terminator v3.1 Cycle Sequencing Kit and 3100 Series Genetic Analyzer (Thermo Fisher Scientific). Electropherograms were analyzed with Sequencher v.10 DNA Software (Gene Codes).

## Results and Discussion

Mutational analysis performed by deep sequencing revealed the presence of a single-nucleotide substitution (c.869-2A>G) in *NIPBL* in both patients. In Individual 1, this variant was found in 23% of the reads (251/1115) in DNA isolated from blood and in 51% of the reads (76/148) in DNA isolated from buccal swab. From Individual 2, only blood DNA was available for the study, and substitution was detected in 46% of the sequencing reads (522/1132). Additional molecular studies performed on the cDNA of both Individuals confirmed that this variant causes aberrant splicing of *NIPBL* transcript, as previously reported (Teresa-Rodrigo et al., 2014). Namely, the intronic variant c.869-2A>G resulted in the in-frame deletion of exon 9 [p.Gly290_Lys498del]. Mutational analysis results are shown in **Figure 1**. Additional molecular analysis could exclude the presence of the *NIPBL* variant in the father of Individual 2 (data not shown).

Here, we report a pathogenic variant detected within a conserved splice-acceptor of *NIPBL* in two family members. Although the variant was previously described as heterozygous germline (Teresa-Rodrigo et al., 2014), we could give experimental evidence of somatic mosaicism in the mother (Patient P3B).

The single nucleotide substitution (c.869-2A>G) within intron 8 of *NIPBL* results in the in-frame deletion of exon 9 and is predicted to cause an altered NIPBL protein lacking 209 amino acids. In Individual 1, the variant was detected in 23 and 51% of sequencing reads in DNA derived from blood and buccal swab samples, respectively. In Individual 2, mutational analysis on blood DNA allowed to detect a substitution in 46% of the reads.

Acuna-Hidalgo et al. (2015) proposed two molecular mechanism responsible for induction of somatic mosaicism: the first as inherited from one of the parents with low-level of mosaicism, a second due to post-zygotic change in the embryo.

We were not able to determine underlying mechanism in Individual 1 since molecular analysis was not performed in her parents. However, both parents were healthy individuals with no physical or intellectual symptoms of CdLS. They both have negative family history of congenital defects. Therefore, with high probability we can omit the inherited form of *NIPBL* alteration.

The more likely mechanism for Individual 1 was a ‘post-zygotic’ mutational event in the embryo. The presence of a genetic alteration in cells from two different germ layers, peripheral blood lymphocytes (mesoderm) and oral mucosa epithelial cells (ectoderm), in mosaic stage indicate that alteration could appear after zygotic stage but before gastrulation process. Because only two tissues were tested, it is difficult to confidently place a time on when and where mosaicism occurred.

In general, various levels of specific alteration in different tissues were previously described as molecular mechanism responsible for phenotypic heterogeneity of CdLS patients (Pozojevic et al., 2017). In Individual 1, it might be possible that low levels of aberrant *NIPBL* in neural tissue could explain the mild intellectual disability. However, these assumptions are contrast to the embryological data. Indeed, the oral epithelium, heterozygous for the variant, derives from embryonic ectoderm which is also the precursor for the neural tube leading to brain development. Thus, different level of alteration in tissues from the same germ layer cannot be excluded, and such a situation could be observed in Individual 1. However, in particular tissues the presence of a high number of mutant cells might not be tolerated and the number of cells carrying the mutation may be determined by how well mutant cells survived and proliferated during development of that tissue, a phenomenon known as negative selection against the mutation (Baquero-Montoya et al., 2014). In patients, the mosaic *NIPBL* alteration led to mild intellectual disability in Individual 1, in comparison to the more severely affected Individual 2.

In Individual 2, the inherited substitution is expressed at the same level in all cells resulting in more severe physical and intellectual manifestations than those observed in his mother.

## Concluding Remarks

Phenotypic variability among CdLS patients, even in the presence of the same type of pathogenic mutation, can be explained by different levels of expression of a genetic variant in tissues from the same or different germ layers. Based on this study, we conclude that at least in some CdLS patients a high sensitivity molecular techniques should be applied and different tissues should be tested to explain the molecular basis of disease in these individuals.

## Ethics Statement

The study was approved by the Ethical Committee of the Medical University of Gdańsk, Poland (NKEBN/395/2014; NKEBN/395-504/2014; and NKEBN/395-288/2014). Written informed consent to participate in the study was obtained from patients’ parents. Written informed consent for publication was obtained from patient’ parents (in the case of Individual 2) and patient (in the case of Individual 1).

## Author Contributions

JW classified the patients in this study. NK and BW conceived and designed the experiments and analyzed the data. NK and AK performed the experiments. NK, AK, IP, JW, BW, and FK prepared the manuscript. All authors read and approved the final manuscript.

## Conflict of Interest Statement

The authors declare that the research was conducted in the absence of any commercial or financial relationships that could be construed as a potential conflict of interest.
